# Assessing fatigue in adults with axial spondyloarthritis: a systematic review of the quality and acceptability of patient-reported outcome measures

**DOI:** 10.1093/rap/rky017

**Published:** 2018-05-29

**Authors:** Nathan A Pearson, Jonathan C Packham, Elizabeth Tutton, Helen Parsons, Kirstie L Haywood

**Affiliations:** 1 Warwick Research in Nursing, Division of Health Sciences, Warwick Medical School, University of Warwick, Coventry, UK; 2 Institute of Applied Clinical Science, Keele University, Staffordshire, UK; 3 Haywood Academic Rheumatology Centre, Staffordshire, UK; 4 Trauma Research, Kadoorie Centre, John Radcliffe Hospital, Oxford University Hospitals, Oxford, UK; 5 Clinical Trials Unit, Warwick Medical School, University of Warwick, Coventry, UK

**Keywords:** fatigue assessment, measurement quality, acceptability, axial spondyloarthritis, systematic review

## Abstract

**Objective:**

The aim was to evaluate the quality and acceptability of patient-reported outcome measures used to assess fatigue in patients with axial spondyloarthritis.

**Methods:**

A two-stage systematic review of major electronic databases (1980–2017) was carried out to: (i) identify measures; and (ii) identify evaluative studies. Study and measurement quality were evaluated following international standards. Measurement content was appraised against a conceptual model of RA-fatigue.

**Results:**

From 387 reviewed abstracts, 23 articles provided evidence for nine fatigue-specific measures: 6 multi-item and 3 single-item. No axial spondyloarthritis-fatigue-specific measure was identified. Evidence of reliability was limited, but acceptable for the Multi-dimensional Fatigue Inventory (internal consistency, test–retest) and Short Form 36-item Health Survey Vitality subscale (SF-36 VT; internal consistency). Evidence of construct validity was moderate for the Functional Assessment of Chronic Illness Therapy-Fatigue and 10 cm visual analog scale, limited for the SF-36 VT and not available for the remaining measures. Responsiveness was rarely evaluated. Evidence of measurement error, content validity or structural validity was not identified. Most measures provide a limited reflection of fatigue; the most comprehensive were the Multi-dimensional Assessment of Fatigue, Multi-dimensional Fatigue Inventory-20, Functional Assessment of Chronic Illness Therapy-fatigue and Fatigue Severity Scale.

**Conclusion:**

The limited content and often poor quality of the reviewed measures limit any clear recommendation for fatigue assessment in this population; assessments should be applied with caution until further robust evidence is established. Well-developed, patient-derived measures can provide essential evidence of the patient’s perspective to inform clinical research and drive tailored health care. The collaborative engagement of key stakeholders must seek to ensure that future fatigue assessment is relevant, acceptable and of high quality.

**Key messages**

- Fatigue is important to patients, but the quality and acceptability of assessment are limited.
- Fatigue assessment is limited by methodological quality and limited relevance to patients.
- Future guidance should be co-produced with patients, ensuring both assessment relevance and methodological rigor.

## Introduction

Pain, stiffness, reduced mobility and fatigue are cardinal features of axial spondyloarthritis (axSpA), including AS [1]. However, despite the importance afforded to fatigue by patients [2, 3], fatigue severity was added to international assessment guidance for axSpA only in 2009 [4]. Accordingly, fatigue assessment in axSpA clinical trials increased significantly from a mere 17.1% of trials completed pre-2001 to 84% post-2001 [5], with most trials (84%) using the single fatigue-severity visual analog scale (VAS) recommended in the assessment guidance [6]. A recent conceptualization of fatigue in RA demonstrated the multifaceted and often complex relationships between disease-specific, cognitive/behavioural (behaviour, cognitive, emotion) and personal (support, health, environment, responsibilities) factors [7]; a complexity that might not be readily captured with a single item of severity [8]. Moreover, individuals experiencing significant impairment owing to frequent, but not severe (VAS scores <5), fatigue would not be identified if assessment were informed purely by fatigue severity [8]. Patients’ fatigue experience may, therefore, be better captured with multi-item, multidomain patient-reported outcome measures (PROMs), providing a structured, patient-reported assessment of health [9, 10]. These may be generic, containing items reflecting general health and completed by any population, or specific to a condition (e.g. axSpA), an aspect of health (e.g. fatigue) or a population (e.g. children). A scoping review of fatigue measures used in rheumatology listed >12 multi-item measures, but only one rheumatology-specific, multi-item measure [11], the Bristol RA Fatigue Multi-Dimensional Questionnaire [12, 13]. However, the quality, acceptability and relevance of measures was not explored, thus limiting evidence-based recommendations.

This review will systematically appraise, compare and synthesize published evidence of the quality and acceptability of clearly defined single- and multi-item PROMs used in fatigue assessment in axSpA to establish the quality and acceptability of fatigue measures. The review will provide a transparent assessment of the evidence with which to inform PROM selection for future application in axSpA research and clinical practice.

## Methods

### Identification of studies and PROMs

Medical subject headings and free text searching reflected: (i) population: axSpA/AS; (ii) construct: fatigue; (iii) assessment type: PROMs; and (iv) measurement and practical properties [14]. Five databases were searched: Medline (OVID), Embase (OVID), PsycINFO (OVID), Cumulative Index of Nursing and Allied Health Literature and Web of Science; from January 1980 to August 2017. A second search used the names of identified measures: (i) population; (ii) construct; (iii) named measures; and (iv) measurement properties (supplementary Appendix S1). Reference lists of included studies and existing reviews were reviewed [11, 15].

### Eligibility criteria

One author (N.A.P.) assessed all titles and abstracts; agreement was independently checked on a 10% subset by a second author (K.L.H.). A third author (J.C.P.) double-assessed all abstracts relating to PsA. Any conflicts were resolved through discussion.

#### Study inclusion

Studies were included if they contained a clearly identifiable and reproducible patient-reported assessment of fatigue, reported evidence of development and/or evaluation after completion by axSpA patients, and were written in English. Studies were excluded if they were available only as abstracts, fatigue assessment was not patient reported, clearly identifiable or reproducible, or the study described PROM application only.

#### PROM inclusion

PROMs were included if they were fatigue specific, assessed fatigue as a separate domain within a multidomain measure, or were single or multi-item assessments. Clinician-reported assessments were excluded.

### Data extraction and appraisal

Data extraction was informed by earlier published reviews [16–19], and the COnsensus-based Standards for the selection of health status Measurement INstruments (COSMIN) checklist [20–22]. Study and PROM-specific information was extracted. Evidence of measurement properties included: validity, reliability, responsiveness and interpretability (supplementary Appendix S2, available at *Rheumatology Advances in Practice* online). Practical properties included evidence of feasibility (administration time; scoring) and acceptability (patient relevance). Evidence of fatigue conceptualization and information pertaining to patient involvement was extracted and recorded. The RA-fatigue conceptual model [7] informed a comparative appraisal of PROM item content. One reviewer (N.A.P.) completed all data extraction. A 10% subset was independently double-extracted (K.L.H.) and agreement checked.

### Assessment of study methodological quality

The COSMIN four-point checklist informed an assessment of study methodological quality for each reported measurement property: poor, fair, good or excellent [20–22]. The lowest item rating per measurement property informed the overall score.

### Assessment of PROM quality

A synthesis of recommendations described by others [18, 19, 23] facilitated the transparent appraisal of PROM quality. Measurement properties were appraised and rated accordingly: adequate (+); inadequate (−); conflicting (±) or unclear (?) (supplementary Appendix 2, available at *Rheumatology Advances in Practice* online).

### Data synthesis and PROM recommendation

Four factors informed the synthesis: (i) study methodological quality (COSMIN); (ii) number of studies reporting evidence; (iii) ratings for measurement/practical properties per measure; and (iv) consistency of results between studies [16, 18]. The final synthesis, hence the evidence upon which PROM recommendation will be made, reflects both: (i) the quality of each measurement property: adequate (+), not adequate (−), conflicting (±) or unclear (?) (supplementary Appendix S2, available at *Rheumatology Advances in Practice* online); and (ii) the overall level of evidence for each measurement property: ‘strong—consistent findings in multiple studies of good methodological quality OR in one study of excellent quality’, ‘moderate—consistent findings in multiple studies of fair methodological quality OR in one study of good quality’, ‘limited—one study of fair methodological quality’, ‘conflicting—conflicting findings’ or ‘unknown—only studies of poor methodological quality’ [18].

PROM recommendations will consider: (i) the extent to which key domains of fatigue identified in the RA-fatigue model are reflected in the PROM (content validity); (ii) whether there is adequate evidence, minimally, of measurement validity (structural and construct) and reliability (internal consistency and test–retest); and (iii) an evidence base that is judged, as a minimum, to be moderate.

## Results

### Identification of studies and PROMs

**Fig. 1:**
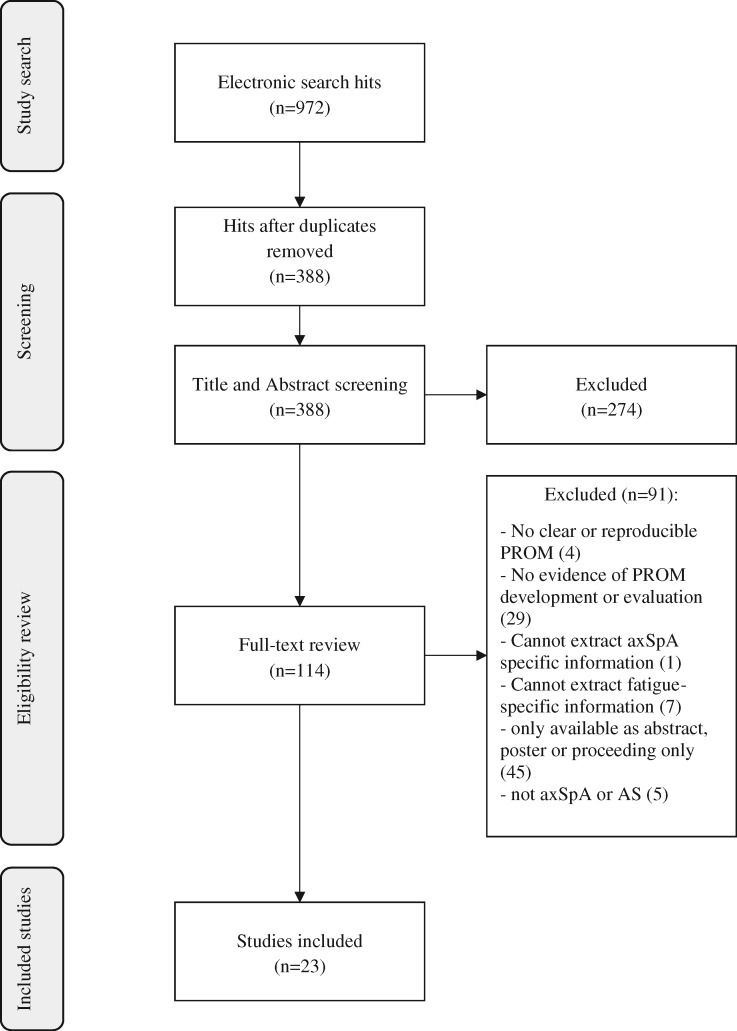
PRISMA flow-chart of study inclusion

A PRISMA flowchart summarizes the review process (Fig. 1). Twenty-three articles provided evidence for nine fatigue-specific PROMs (Table 1). There were three multidimensional fatigue-specific PROMs: Multi-dimensional Assessment of Fatigue (MAF) [24], Multi-dimensional Fatigue Inventory (MFI-20) [25] and Multi-dimensional Fatigue Symptom Inventory–Short Form (MFSI-SF) [26]; three uni-dimensional: Functional Assessment of Chronic Illness Therapy (Fatigue) (FACIT-fatigue) [27], Fatigue Severity Scale (FSS) [28] and the vitality subscale (VT) of the Short-Form 36-item Health Status Survey (SF-36) [29]; and three single-item questions: Worst-Fatigue Numeric Rating Scale (WF-NRS) from the Brief Fatigue Inventory (BFI) [30], the 10 cm fatigue severity VAS (from the BASDAI) [31] and a modified 10 cm VAS whereby the descriptor ‘none’ was changed to ‘no problem’ [32].

**Table 1:** Summary of the reviewed single- and multi-item fatigue PROMs (*n *=* *9)

PROM	Conceptual focus	How to score
References	Response options; recall period	
Completion format		
Completion time		
**Multidimensional fatigue measures (3/9)**		
Multi-dimensional Assessment of Fatigue (MAF) Tack *et al.* [24] Completion format: self-completed Completion time: ∼<10 min	A revision of the Piper Fatigue Scale (PFS), which was developed and tested with oncology patients and was designed to measure: temporal, severity and sensory fatigue dimensions. The 41-item PFS was reduced to form the 16-item MAF. The MAF was developed to measure four dimensions: fatigue severity, distress, impact and timing. No information provided about item selection, retention or generation. No evidence of patient involvement as research partners. MAF developed to measure multiple dimensions of fatigue in adult RA patients Fifteen questions, which contribute to a global fatigue index (GFI). A 16th question does not contribute to the GFI. Four subscales explored: distress, severity, interference in daily living activities and frequency/change during the last week Response options NRS from 1 to 10. Anchors vary by items Items 1 and 4–14 are anchored with ‘not at all’ and ‘a great deal’ Item 2 is anchored with ‘mild’ and ‘severe’ Item 3 is anchored with ‘no distress’ and ‘a great deal of distress’ Items 15 and 16 require an ordinal response with four options scored 1–4 Item 15 is anchored with ‘hardly any days’ to ‘every day’ Item 16 is anchored with ‘decreased’ to ‘increased’ Recall period 1 week	If item 1 is scored as 0 then the remaining items should be scored as 0 Calculating the GFI should be done using the following four steps: (i) sum items 1–3; (ii) average items 4–14 (do not include activities marked as ‘do not do’ in the average; (iii) multiply item 15 by 2.5 to create a score from 0 to 10; (iv) sum the values from (i)–(iii) together to obtain a GFI Item 16 does not contribute to the GFI but is scored from 1 to 4
Multi-dimensional Fatigue Inventory (MFI-20) Smets *et al.* [25] Completion format: self-completed Completion time: ∼5 min	Developed to measure fatigue in cancer patients without somatic items. Initial development was informed by the authors and previous research, resulting in five proposed domains: general, physical sensations and cognitive symptoms, which were theoretically supported for inclusion in the MFI based on factor analyses conducted in other fatigue studies. Reduced motivation and reduced activity formed the final two components, but it is unclear whether reduced activity was considered a consequence of fatigue. No information about the process of item generation. Patients were included as participants but not research partners. Initially evaluated in patients with cancer, chronic fatigue syndrome and healthy individuals who may experience physical fatigue (military personnel) or mental fatigue (newly qualified doctors) Five subscales, each made up of four items (total = 20 items). Half of the items are positively phrased, thus requiring reverse scoring. The following five subscales were explored: general fatigue, physical fatigue, reduced activity, reduced motivation and mental fatigue Response options A total of five check boxes, with anchors ‘yes that is true’ to ‘no that is not true’ Recall period No specific time scale stated. Instructions state ‘…how you have been feeling lately…’	Each item is scored from 1 to 5. Positively phrased questions (*n* = 10) must be reverse scored Subscales can be individually scored by summing their respective items
Multi-dimensional Fatigue Symptom Inventory—Short Form (MFSI-SF) Stein *et al.* [26] Completion format: self-completed Completion time: ∼5 min	MFSI-SF is derived from the MFSI, which is an 83-item measure informed by evidence from fatigue literature, discussions with experts who treat fatigue patients and a review of ‘current’ fatigue measures. Subscales were empirically derived from factor analysis. Five domains were identified: (i) global fatigue experience; (ii) somatic symptoms; (iii) cognitive symptoms; (iv) affective symptoms; and (v) behavioural symptoms. Patients were included as participants but not as research partners. The MFSI-SF is made up of the empirically derived subscales only. Developed for use in cancer patients Five subscales, each made up of six items (total = 30 items). The five subscales explore: general fatigue, physical fatigue, emotional fatigue, mental fatigue and vigour Response options Five response options ranging from 0 to 4, with anchors ‘not at all’ to ‘extremely’ Recall period Past 7 days	Total score is a summation of four subscales (general + physical + emotional + mental) minus vigour
**Unidimensional fatigue measures (2/9)**		
Functional Assessment Chronic Illness Therapy (FACIT-fatigue) Yellen *et al.* [27] Completion format: self-completed Completion time: ∼<5 min	FACIT-fatigue was developed to measure cancer-related fatigue. Development was two phase: phase 1 (item generation) used semi-structured interviews with cancer patients and medical experts; phase 2 (item reduction) was a presentation of questions to a second group of medical experts and their review. No conceptual model of fatigue was reported. Patients were included as participants but not research partners. Initial psychometric evaluation (with cancer patients) indicates that the measure has good validity and reliability Measure is made up of 13 items that ask about fatigue and how it impacts on daily activities Response options Five response options, with anchors ‘not at all’ to ‘very much’ Recall period Past 7 days	Global score calculated by summing item scores
Fatigue Severity Scale (FSS) Krupp *et al.* [28] Completion format: self-completed Completion time: ∼<5 min	Developed to assess fatigue in patients with multiple sclerosis and systematic lupus erythematosus. Initial 28-item fatigue questionnaire was reduced based on the results of a factor analysis, item analysis and theoretical considerations; unclear what these considerations were. Five judges sorted items, without labels, into domains. No conceptual model of fatigue was reported. Patients were included in the study as research participants only, and not as research partners Measure made up of nine items and an additional ‘global fatigue’ VAS Response options Seven response options ranging from 1 to 7 with anchors ‘strongly disagree’ to ‘strongly agree’ Recall period Past week	Global score calculated by summing item scores and then averaging
**Single-item fatigue measures (3/9)**		
Brief Fatigue Inventory (BFI) Worst Fatigue Numeric Rating Scale (WF-NRS) Mendoza *et al.* [30] Completion format: self-completed Completion time: approximately <2 min	The BFI was developed to assess fatigue in cancer patients and the impact over the past day. The BFI was based on the Brief Pain Inventory (BPI). Questionnaire items were modified following revision of ‘a fatigue questionnaire’ completed by cancer patients and healthy controls in a previous study. Partial conceptualization was provided in the explanation of item revision, but the information was limited. Questions seek to investigate: fatigue severity, interference with function; factors that worsen fatigue; and contributing factors to fatigue. It consists of 10 items across two subscales: Fatigue Severity (four items); and Fatigue Impact (six items). Patients were involved as participants, but there was no evidence reported that they were involved as research partners The WF-NRS is a single-item on fatigue severity Response options WF-NRS (item 3) ranges from 0 to 10, with anchors ranging from ‘no fatigue’ to ‘as bad as you can imagine’ Recall period Past 24 h	Score taken from the value marked on the VAS
BASDAI 10 cm VAS Garrett *et al.* [31] Completion format: self-completed Completion time: ∼<2 min	The BASDAI is an AS-specific measure of disease activity. Development was driven by a team of physiotherapists, research associates and rheumatologists. Patients were used as participants to complete a pilot version of the questionnaire, but not as research partners. No conceptual model of fatigue was reported. The six items assess pain (severity), fatigue/tiredness (severity), stiffness (duration and severity) and tenderness (severity) Response options 10 cm VAS, with anchors ‘none’ and ‘very severe’ at either end of the scale Recall period 1 week	Score taken from the value marked on the VAS
Modified 10 cm VAS Wheaton *et al.* [32] Completion format: self-completed Completion time: ∼<2 min	See BASDAI 10 cm VAS Response options 10 cm VAS, with anchors ‘no problem’ and ‘very severe’ at either end of the scale Recall period Not specified; assumed 1 week (as per original BASDAI single-item on fatigue severity)	Score taken from the value marked on the VAS
**Fatigue-specific PROM subscale (1/9)**		
Short Form 36-item Health Survey (SF-36) SF-36 vitality subscale Ware & Sherbourne [29] Completion format: self-completed Completion time: ∼<5 min	The SF-36 is a generic measure of health status, containing 36-items across eight health domains: physical functioning, role limitations owing to physical health or emotional problems, energy, pain, emotional wellbeing, social role functioning and general health. Most of the items included were taken from established measures. The content of these was reviewed with a view to assign the content to the pre-defined domains; these were informed by data from a previous iteration of the short form health survey (SF-20). Patients were not involved as research partners in the development process Vitality subscale made up of four items (two positively, two negatively phrased) Response options Six response options, with anchors ‘none of the time’ and ‘all of the time’ Recall period Last 4 weeks	Positively phrased questions must be reverse scored. Scores then summed for all four items

PROM: patient-reported outcome measure; VAS: visual analog scale.

### Study and sample characteristics

All studies included adults with a primary diagnosis of axSpA, aged between 18 and 72 years old (supplementary Appendix S3, available at *Rheumatology Advances in Practice* online). Sample sizes ranged from 40 to 812. Studies were predominantly cross-sectional, investigating fatigue prevalence and/or its association with other variables.

### Measurement properties and methodological quality

Study methodological quality (per PROM) was assessed and recorded (supplementary Appendix S4, available at *Rheumatology Advances in Practice* online). An evidence synthesis is presented in Table 2. Evidence of measurement error, content or structural validity, criterion-based responsiveness, acceptability or feasibility of completion was not identified.

**Table 2:** Data synthesis^a^, levels of evidence and overall quality^b^ of reviewed PROMs (*n *=* *9)

PROM/study	Number of evaluations	Reliability		Construct validity		Responsiveness		Interpretation
		Internal consistency	Reliability	Hypothesis testing	Known groups	Responsiveness	Other	
**Multidimensional fatigue measures (3/9)**								
MAF	7				?			
					Unknown			
MFI-20	2	+	+	?	?		ES, SRM, Guyatt	
		Limited	Limited	Unknown	Unknown			
MFSI-SF	1			?	?			
				Unknown	Unknown			
**Unidimensional fatigue measures (2/9)**								
FACIT-fatigue	2	?		+		?		
						Unknown		
		Unknown						
				Moderate				
FSS	3			?	?		ES, SRM	
				Unknown	Unknown			
**Single-item fatigue measures (3/9)**								
BFI WF NRS	1	Review of practical properties only						
10 cm VAS (BASDAI)	18		−	+	?	+	ES, SRM, Guyatt	?
			Limited	Moderate	Unknown	Limited		Unknown
Modified 10 cm VAS	1							?
								Unknown
**Fatigue-specific PROM subscale (1/9)**								
SF-36(vitality domain)	10	+		+	?		ES, SRM	
		Moderate		Limited	Unknown			

The synthesis considered the following four factors: (i) study methodological quality (COSMIN scores); (ii) number of studies reporting evidence of measurement properties (per PROM); (iii) results for each measurement property (per PROM); and (iv) consistency of results between studies. The results of data synthesis include the following two ratings: (i) overall measurement property quality: adequate (+), not adequate (−), conflicting (±) or unclear (?); and (ii) levels of evidence for the overall quality per measurement property. Five outcomes were defined: strong: consistent findings in multiple studies of good methodological quality or in one study of excellent quality; moderate: consistent findings in multiple studies of fair methodological quality or in one study of good methodological quality; limited: one study of fair methodological quality; conflicting: conflicting findings; or unknown evidence: only studies of poor methodological quality [18, 23].

^a^ Data synthesis: qualitative synthesis of the data determined the quality and acceptability of each reviewed PROM.

^b^ Overall quality: There was no measurement evidence available for the following measurement properties, and they are therefore not referred to in the synthesis table: measurement error, content validity and structural validity. BFI: Brief Fatigue Inventory; COSMIN: COnsensus-based Standards for the selection of health Measurement Instruments; ES: effective size; FACIT-fatigue: Functional Assessment of Chronic Illness Therapy-fatigue; FSS: Fatigue Severity Scale; MAF: Multi-dimensional Assessment of Fatigue; MFI: Multi-dimensional Fatigue Inventory; MFSI-SF: Multi-dimensional Fatigue Symptom Inventory-Short Form; PROM: Patient reported outcome measure; SF-36: Short Form 36-item Health Survey; SRM: standardised response mean; VAS: visual analogue scale WF-NRS: Worst Fatigue-Numeric Rating Scale.

### Fatigue conceptualization and patient involvement

A review of PROM development suggests very limited conceptualization of fatigue for four PROMs (MFI-20, MFSI-SF, SF-36 and BFI; Table 1). Item generation or selection was often poorly reported and lacking in transparency. Only the single-item VAS of fatigue severity (taken from the BASDAI) was developed specifically for use with axSpA patients, but a conceptualization of fatigue was absent. The involvement of patients did not extend beyond participation (i.e. simply measurement completion); no study included patients as research partners in measurement evaluation.

### Comparative item content

Although similarities of item content exist, all reviewed measures provided a limited reflection of the RA-fatigue model (Table 3). All single-item measures assessed fatigue severity.

**Table 3:** Item content of the reviewed single- and multi-item fatigue measures (*n* = 9): item content distribution as per the Hewlett conceptual model of RA fatigue [7]

PROM	Concepts of RA fatigue								
	Disease specific	Cognitive, behavioural			Personal				Symptoms
		Behaviour	Cognition	Emotion	Support	Health	Environment	Responsibilities	
**Multidimensional fatigue measures (3/9)**									
MAF		Physical impact (2); ability to do chores in house, exercise Physical leisure activities (2); sex, recreational activities Physical effect on daily activities (5); cook, bathe, dress, walk, shop/errands		Emotional impact (1); distress				Interference with social life (1) and work (1)	Severity (1) Frequency (1) General (1); extent of experiencing fatigue
MFI-20		Limitation (1); physically feel I can only do a little Capability (1); physically I feel I can take a lot on Activity level (1); I get little done	Cognition (4); focus, concentration Forethinking (1); lots of plans Motivation (1); do not feel like doing anything	Anxiety (1); dread		Self-perception (10); I am rested, physically I am in bad condition, I tire easily, physically I am in excellent condition			
MFSI-SF			Memory (2); trouble remembering, forgetful Concentration/focus (4); confused, paying attention, unable to concentrate, making mistake, forgetful	Mood (9); upset, cheerful, nervous, relaxed, sad, depressed, tense, calm, distressed		Symptom manifestation (12); aching, weak, tired, heavy, pooped, fatigued Self-perception (3); feel: lively, refreshed, energetic			
**Unidimensional fatigue measures (2/9)**									
FACIT-fatigue		Ability (1); able to do usual activities Impairment (1); I need help to do my usual activities Social activity (1); have to limit because tired	Motivation (2); trouble starting, trouble finishing	Mood (1); frustrated by being too tired		Impact (2); need to sleep, too tired to eat Symptom manifestation (4); fatigued, weak, listless, tired Energy (1); I have energy			
FSS		Impact (2); interferes with functioning, prevents sustained physical functioning	Motivation (1); reduced motivation Reflection (1); most disabling symptom					Interference (1); work, family or social life	Generic (1); easily fatigued? Global perception (1); what number best reflects global fatigue Frequency (1); causes frequency problems Causes of fatigue (1); exercise brings on fatigue
**Single-item fatigue measures (3/9)**									
BFI WF-NRS									Severity (1)
10 cm VAS									Severity (1)
Modified 10 cm VAS									Severity (1)
**Fatigue-specific PROM subscale (1/9)**									
SF-36 VT (vitality subscale)						Sense of energy/fatigue (4); full of pep? A lot of energy? Feel worn out? Feel tired			

PROM: patient-reported outcome measure; VAS: visual analog scale.

### Multidimensional fatigue-specific PROMs

#### *MAF [* 24 *]*

Six poor-quality studies provided limited evidence of construct validity (correlations and known-groups validity), including small to moderate associations between the MAF total and AS-specific Bath measures (range 0.23–0.73), and the MAF subscales and SF-36 VT (range 0.3–0.53) and 10 cm fatigue-severity VAS (range 0.39–0.53) [33–38]; all evaluations lacked *a priori* hypothesized associations.

#### *MFI-20 [* 25 *]*

One poor-quality study provided limited evidence of construct validity [39]. A fair-quality study provided acceptable evidence of internal consistency {Cronbach’s α from 0.68 [Reduced Motivation (RM) subscale] to 0.86 [Reduced Activity (RA) subscale]} and construct validity [40] {moderate to strong associations between subscales [general fatigue with physical fatigue (PF) 0.69/RA 0.52/RM 0.45/mental fatigue (MF) 0.45; MF with PF 0.40/RA 0.42/RM 0.48; RM with PF 0.51/RA 0.54] supporting assumed *a priori* hypothesis associations} [40]. Limited evidence for 1-week test–retest reliability was also reported for patients after completion of a VAS on a person’s overall perceived health, taken from the EuroQoL (EQ-5D) (intraclass correlation coefficient, ICC range: PF 0.57–0.75 RM/MF) in a study judged to be of fair quality [39]; for three subscales (GF, PF and RA) values <0.70 were reported. Distribution-based measures of responsiveness [both effect size (ES) statistics and the standardized response mean (SRM)] were calculated from trial data, without any *a priori* hypotheses, following 3-month completion after the end of spa therapy: small values (<0.3) for domains reflecting reduced activity to large (>0.82) for domains reflecting general fatigue and PF were reported (ES: GF 0.82/PF 0.81/RA 0.28/RM 0.54/MF 0.38; SRM: GF 0.70/PF 0.82/RA 0.23/RM 0.51/MF 0.49; Guyatt statistics: GF 0.86/PF 0.96/RA 0.30/RM 0.50/MF 0.57).

#### *Multi* *-* *dimensional Fatigue Symptom Inventory* *—* *Short Form (MFSI-SF) [* 26 *]*

One poor-quality study provided limited evidence of construct validity [41]. Weak to strong associations between the MFSI-SF subscales and the BASDAI 10 cm VAS were reported (10 cm VAS with GF 0.71/PF 0.74/emotional fatigue 0.56/MF 0.45/Vigor −0.32) after completion by 62 AS patients. Although association between variables could be assumed, *a priori* hypothesized associations were not stated.

### Unidimensional fatigue PROMs

#### *FACIT-fatigue [* 27 *]*

One poor-quality study provided acceptable evidence of internal consistency (Cronbach’s α 0.82/0.86), item-level performance (corrected item-total correlation: 0.56/0.88) and construct validity [42]. Good-quality evidence of construct validity was available from the same article. Strong associations were reported between the FACIT-fatigue and SF-36 VT (range *r* = 0.74–0.82) and the 10 cm VAS (*r* = −0.69), with moderate associations with the BASDAI index score (*r* = −0.47) and BASFI (*r* = −0.56) [42]. These findings confirmed *a priori* hypothesized associations between variables.

#### *FSS [* 28 *]*

Both strong (0.77) [43] and moderate (0.53) [44] associations between the FFS and the 10 cm fatigue-severity VAS have been reported in two studies judged to be of poor quality. Small ES were reported at 28 days for participants in both arms of a placebo-controlled trial of s.c. etanercept (ES 0.15/−0.23; SRM 0.22/0.22) [45].

#### *SF-36 vitality subscale [* 29 *]*

One fair-quality study provided acceptable evidence of construct validity [42]: a strong association between the VT subscale and the FACIT-fatigue was reported (*r* = 0.74; *r* = 0.82), a moderate association with the 10 cm VAS (*r* = −0.49) and a weak association with the BASFI (*r* = −0.33). One good-quality study provided acceptable evidence of internal consistency and item-level performance (Cronbach’s α 0.78/0.88; item-total correlation 0.57/0.64) [42]. Moderate to large ES statistics were reported at both 28 days (ES = 0.54; SRM = 0.83) and 112 days (ES = 0.69; SRM = 0.75) in patients receiving 25 mg of etanercept s.c., twice weekly [45].

### Single-item fatigue PROMs

#### *10 cm fatigue-severity VAS [* 31 *]*

One good-quality study provided acceptable evidence of construct validity [42]. A strong association between the item and the FACIT-fatigue (*r* = −0.69), and a moderate association with the SF-36 VT (*r* = −0.49) was reported after completion by AS patients participating in a double-blind, placebo-controlled clinical trial, supporting *a priori* hypothesized associations.

A level of test–retest reliability judged to be below accepted standards for group analysis (ICC = 0.60) was reported after a 6-week test–retest period in patients defined as stable on the EuroQoL EQ-VAS (general health); the study was judged to be of fair quality [40]. However, estimates for test–retest reliability were below accepted thresholds for use with groups (0.70) or individuals (0.90) [47]. In comparison with participants who received placebo or NSAIDs (small ES −0.35) [46], large ES statistics (ES = 0.89; SRM = 0.89; Guyatt statistics 0.92) were reported at 6 weeks for participants receiving the active, spa therapy intervention [40].

#### *Modified 10 cm VAS [* 32 *]*

The 10 cm fatigue-severity VAS descriptor none was modified to no problem, changing the response scale. One poor-quality study provided limited, poor-quality interpretative guidance [32].

#### *BFI* *—* *WF-NRS [* 30 *]*

One qualitative study explored the relevance and acceptability of the WF-NRS single item taken from the BFI [48]. Although the item was judged to be relevant, the phraseology was confusing (‘what best describes your *worst* fatigue’). A longer recall period than 24 h was also recommended, to express fatigue variability better.

## Discussion

Greater understanding of the impact of fatigue has been identified as a priority by axSpA patients [3]. However, current assessment guidance is limited to a single-item measure of fatigue severity [4], which underestimates the often profound and wide-ranging impact of fatigue on an individual’s life. Of the nine reviewed measures, only three were multidimensional, containing items reflecting different aspects of fatigue. However, no measure was specific to the experience of axSpA-fatigue and none had been evaluated for its relevance to axSpA patients. There was limited and often poor-quality evidence of reliability and construct validity; and an absence of interpretative guidance and evidence of measurement error, content validity or structural validity for any of the reviewed measures. Evidence of responsiveness was limited to the reporting of effective size statistics, which fail to provide an accurate evaluation of the ability of a measure to detect meaningful change in health [19]. Consequently, the lack of minimal measurement evidence for validity and reliability means that it is not possible to make any assessment recommendations.

This is the first review of the quality and acceptability of measures of fatigue after completion by patients with axSpA. The results are strengthened by an evaluation of both study [20, 21] and PROM quality [16, 18, 19, 23], paired with a detailed comparative appraisal of item content. However, much of the extracted data came from studies where PROM evaluation was not the primary focus of the study. As such, the rigour of the COSMIN criteria meant that these studies typically scored poorly. Although a single reviewer (N.A.P.) assessed all titles and abstracts for review eligibility, a sub-set of titles and abstracts were reviewed by a second reviewer (K.L.H.) and reliability was checked.

Adoption of the RA-fatigue conceptual model in the present review highlighted the limited content validity of the reviewed measures. No PROM fully reflects the RA-model of fatigue. Both the MAF and the FSS include the assessment of fatigue frequency and severity, two important components of the fatigue experience for axSpA patients [8]. However, only two PROMs {the MFI-20 [10/20 (total) items] and FACIT-fatigue (6/13 items)} include items that seek to assess the cognitive/behavioural (and emotional) impact of fatigue. Other PROMs (MAF, MFSI-SF and FSS) include items limited to only two of the cognitive/behavioural domains. Although adequate evidence of internal consistency and reliability was reported for the MFI-20, it is unclear whether the PROM can detect change, or if it measures components of fatigue important to axSpA patients. Acceptable, but limited, evidence of a strong association between the FACIT-fatigue and SF-36 VT enhances confidence in the ability of the FACIT-fatigue to measure fatigue in this population. However, evidence of measurement reliability and responsiveness is lacking in the axSpA population. Consequently, although demonstrating acceptable item content, both measures lack acceptable evidence of essential psychometric properties currently to support their use in axSpA-fatigue assessment. A robust fatigue assessment is necessary to detect and detail the nuances of fatigue experience that are essential to providing individualized and tailored health care to axSpA patients.

Qualitative research has detailed a similar experience of fatigue in axSpA, highlighting the significant impact of fatigue on social life, patient mental health and relationships with others, their ability to engage with usual activities of daily living [49] and their reliance on self-management strategies [2]. This demonstrates the importance of considering these aspects in the assessment of fatigue impact, and the insufficient information available from using only a single-item VAS of fatigue severity [49]. Similarities between RA and axSpA-fatigue experience support the appropriateness of the RA-fatigue model as a framework against which PROM content and relevance can be appraised for use with the axSpA population [49, 50]. However, growing evidence demonstrates that fatigue experience is a dynamic, complex and multifaceted experience that is, to a large extent, disease specific. For example, evidence has shown both similarities and differences in fatigue experience between related and unrelated conditions (FM, multiple sclerosis, AS and stroke) [51] and between different stages of illness, such as patients with active cancer compared with cancer survivors [52]. Therefore, although this review has used the RA-fatigue model to appraise PROM item content, it is essential that a conceptual model is developed to reflect the nuances specific to the experience and impact of axSpA-fatigue.

A review of the quality of fatigue measures used in a range of chronic illnesses also highlighted the lack of evidence of essential measurement properties, thus limiting recommendations [53]. However, the judgement of measurement quality lacked transparency, and study methodological quality was not determined. International guidance promotes the importance of greater transparency in the assessment of measurement quality and acceptability [19, 23, 54]. Adoption of the COSMIN checklist, as in the present review, facilitates the incorporation of study methodological quality in the final judgement of PROM quality [20–22].

Well-developed, patient-derived PROMs are both robust and relevant to the experience of patients, capturing the outcomes that really matter [10, 55]. However, numerous legacy measures, where content was largely driven by the perspective of clinicians, may lack relevance to patients [10, 55]. The failure of PROMs to capture the outcomes that really matter to patients [9, 56–58] undermines the potential contribution to patient-centred care and shared decision-making, and was the driver for the co-development of a new, patient-derived measure of fatigue for RA, namely the Bristol RA Fatigue Multi-dimensional Questionnaire [12, 13]. Of the nine PROMs identified in this review, only four provided a limited conceptualization of fatigue, which was mostly derived from literature reviews and clinical experts. Only one PROM (FACIT-Fatigue) was developed following a qualitative method (semi-structured interviews) but did not provide a conceptualization of fatigue. Qualitative research offers greater insight into key health issues affecting patients, improving the relevance and acceptability of PROM content. This can highlight the unmet needs of patients, supporting targeted health-care efforts to address what really matters to the patient.

The MFI-20 and FACIT-fatigue provide the most comprehensive assessment of fatigue [7], but evidence of their psychometric qualities in the axSpA population is limited.

A limited number of fatigue-specific PROMs have been evaluated for their quality and acceptability for use in axSpA fatigue assessment. However, recommendations are limited by the poor methodological quality of most studies coupled with the limited evidence of robust measurement or practical properties. These limitations also suggest that data generated from the application of these measures in routine practice or clinical research settings should be interpreted with caution. A comparative appraisal of PROM content suggests that the MFI and FACIT-fatigue provide the most comprehensive assessment of fatigue, including the impact on both cognition and behaviour. However, further exploration of the relevance and acceptability of the reviewed measures to patients with axSpA-fatigue is warranted. Moreover, comparative evaluations of those measures that have acceptable content validity are urgently required to establish robust evidence of essential measurement properties; specifically, reliability, validity, responsiveness and interpretation.

*Funding statement*: This work was supported by the National Ankylosing Spondylitis Society (NASS) [grant number: WAR1].

*Disclosure statement*: The authors declare no conflict of interest.

## Supplementary data

Supplementary data are available at *Rheumatology Advances in Practice* online.

## Supplementary Material

Supplementary Data

## References

[1] Raine C, Keat A. Axial spondyloarthritis. Medicine 2014;42:251–6.

[2] Davies H, Brophy S, Dennis M et al Patient perspectives of managing fatigue in Ankylosing Spondylitis, and views on potential interventions: a qualitative study. BMC Musculoskelet Disord 2013;14:163. 23659344 10.1186/1471-2474-14-163 PMC3668149

[3] National Ankylosing Spondylitis Society. NASS Research Priorities 2013–2018. 2013 http://nass.co.uk/research/nass-research-priorities/ (11 December 2017, date last accessed).

[4] Sieper J, Rudwaleit M, Baraliakos X et al The Assessment of SpondyloArthritis international Society (ASAS) handbook: a guide to assess spondyloarthritis. Ann Rheum Dis 2009;68(Suppl 2):ii1. 10.1136/ard.2008.104018 19433414

[5] Bautista-Molano W, Navarro-Compán V, Landewé RBM et al How well are the ASAS/OMERACT Core Outcome Sets for Ankylosing Spondylitis implemented in randomized clinical trials? A systematic literature review. Clin Rheumatol 2014;33:1313–22. 24970597 10.1007/s10067-014-2728-6

[6] van der Heijde D, Calin A, Dougados M et al Selection of instruments in the core set for DC-ART, SMARD, physical therapy, and clinical record keeping in ankylosing spondylitis. Progress report of the ASAS Working Group. Assessments in Ankylosing Spondylitis. J Rheumatol 1999;26:951–4. 10229426

[7] Hewlett S, Chalder T, Choy E et al Fatigue in rheumatoid arthritis: time for a conceptual model. Rheumatology 2011;50:1004–6. 20819797 10.1093/rheumatology/keq282

[8] Haywood KL, Packham JC, Jordan KP. Assessing fatigue in ankylosing spondylitis: the importance of frequency and severity. Rheumatology 2014;53:552–6. 24307290 10.1093/rheumatology/ket397

[9] Haywood KL. Patient-reported outcome: measuring what matters or just another paper exercise? Musculoskelet Care 2006;4:63–6. 10.1002/msc.78 17042017

[10] Haywood KL, Wilson R, Staniszewska S, Salek S. Using PROMs in healthcare: who should be in the driving seat—policy makers, health professionals, methodologists or patients? Patient 2016;9:495–8. 27646693 10.1007/s40271-016-0197-5

[11] Hewlett S, Dures E, Almeida C. Measures of fatigue: Bristol Rheumatoid Arthritis Fatigue Multi-Dimensional Questionnaire (BRAF MDQ), Bristol Rheumatoid Arthritis Fatigue Numerical Rating Scales (BRAF NRS) for severity, effect, and coping, Chalder Fatigue Questionnaire (CFQ), Checklist Individual Strength (CIS20R and CIS8R), Fatigue Severity Scale (FSS), Functional Assessment Chronic Illness Therapy (Fatigue) (FACIT-F), Multi-Dimensional Assessment of Fatigue (MAF), Multi-Dimensional Fatigue Inventory (MFI), Pediatric Quality Of Life (PedsQL) Multi-Dimensional Fatigue Scale, Profile of Fatigue (ProF), Short Form 36 Vitality Subscale (SF-36 VT), and Visual Analog Scales (VAS). Arthritis Care Res 2011;63 (Suppl 11):S263–86. 10.1002/acr.20579 22588750

[12] Nicklin J, Cramp F, Kirwan J, Urban M, Hewlett S. Collaboration with patients in the design of patient-reported outcome measures: capturing the experience of fatigue in rheumatoid arthritis. Arthritis Care Res 2010;62:1552–8. 10.1002/acr.20264 20496429

[13] Nicklin J, Cramp F, Kirwan J et al Measuring fatigue in rheumatoid arthritis: a cross-sectional study to evaluate the Bristol Rheumatoid Arthritis Fatigue Multi-Dimensional questionnaire, visual analog scales, and numerical rating scales. Arthritis Care Res 2010;62:1559–68. 10.1002/acr.20282 20583112

[14] Terwee CB, Jansma EP, Riphagen II, de Vet HC. Development of a methodological PubMed search filter for finding studies on measurement properties of measurement instruments. Qual Life Res 2009;18:1115–23. 19711195 10.1007/s11136-009-9528-5 PMC2744791

[15] Haywood KL, Garratt AM, Dawes PT. Patient-assessed health in ankylosing spondylitis: a structured review. Rheumatology 2005;44:577–86. 15695297 10.1093/rheumatology/keh549

[16] Haywood KL, Collin SM, Crawley E. Assessing severity of illness and outcomes of treatment in children with Chronic Fatigue Syndrome/Myalgic Encephalomyelitis (CFS/ME): a systematic review of patient-reported outcome measures (PROMs). Child Care Health Dev 2014;40:806–24. 24661148 10.1111/cch.12135

[17] Haywood KL, Staniszewska S, Chapman S. Quality and acceptability of patient-reported outcome measures used in Chronic Fatigue Syndrome/Myalgic Encephalomyelitis (CFS/ME): a systematic review. Qual Life Res 2012;21:35–52. 21590511 10.1007/s11136-011-9921-8

[18] Elbers RG, Rietberg MB, van Wegen EEH et al Self-report fatigue questionnaires in multiple sclerosis, Parkinson’s disease and stroke: a systematic review of measurement properties. Qual Life Res 2012;21:925–44. 22012025 10.1007/s11136-011-0009-2 PMC3389599

[19] Terwee CB, Bot SD, de Boer MR et al Quality criteria were proposed for measurement properties of health status questionnaires. J Clin Epidemiol 2007;60:34–42. 17161752 10.1016/j.jclinepi.2006.03.012

[20] Mokkink LB, Terwee CB, Patrick DL et al The COSMIN study reached international consensus on taxonomy, terminology, and definitions of measurement properties for health-related patient-reported outcomes. J Clin Epidemiol 2010;63:737–45. 20494804 10.1016/j.jclinepi.2010.02.006

[21] Mokkink LB, Terwee CB, Patrick DL et al The COSMIN checklist for assessing the methodological quality of studies on measurement properties of health status measurement instruments: an international Delphi study. Qual Life Res 2010;19:539–49. 20169472 10.1007/s11136-010-9606-8 PMC2852520

[22] Terwee CB, Mokkink LB, Knol DL. Rating the methodological quality in systematic reviews of studies on measurement properties: a scoring system for the COSMIN checklist. Qual Life Res 2012;21:651–7. 21732199 10.1007/s11136-011-9960-1 PMC3323819

[23] Conijn AP, Jens S, Terwee CB, Breek JC, Koelemay MJ. Assessing the quality of available patient reported outcome measures for intermittent claudication: a systematic review using the COSMIN checklist. Eur J Vasc Endovasc Surg 2015;49:316–34. 25618453 10.1016/j.ejvs.2014.12.002

[24] Tack BB. Dimensions and correlates of fatigue in the older adult with rheumatoid arthritis. PhD Thesis, University of California, San Francisco, 1991.

[25] Smets EM, Garssen B, Bonke B, De Haes JC. The Multidimensional Fatigue Inventory (MFI) psychometric qualities of an instrument to assess fatigue. J Psychosomatic Res 1995;39:315–25. 10.1016/0022-3999(94)00125-o 7636775

[26] Stein KD, Jacobsen PB, Blanchard CM, Thors C. Further validation of the multidimensional fatigue symptom inventory-short form. J Pain Sympt Manag 2004;27:14–23. 10.1016/j.jpainsymman.2003.06.003 PMC2547485 14711465

[27] Yellen SB, Cella DF, Webster K, Blendowski C, Kaplan E. Measuring fatigue and other anemia-related symptoms with the Functional Assessment of Cancer Therapy (FACT) measurement system. J Pain Sympt Manag 1997;13:63–74. 10.1016/s0885-3924(96)00274-6 9095563

[28] Krupp LB, LaRocca NG, Muir-Nash J, Steinberg AD. The fatigue severity scale. Application to patients with multiple sclerosis and systemic lupus erythematosus. Arch Neurol 1989;46:1121–3. 2803071 10.1001/archneur.1989.00520460115022

[29] Ware JE Jr, Sherbourne CD. The MOS 36-item short-form health survey (SF-36). I. Conceptual framework and item selection. Med Care 1992;30:473–83. 1593914

[30] Mendoza TR, Wang XS, Cleeland CS et al The rapid assessment of fatigue severity in cancer patients. Cancer 1999;85:1186–96. 10091805 10.1002/(sici)1097-0142(19990301)85:5<1186::aid-cncr24>3.0.co;2-n

[31] Garrett S, Jenkinson T, Kennedy LG et al A new approach to defining disease status in ankylosing spondylitis: the Bath Ankylosing Spondylitis Disease Activity Index. J Rheumatol 1994;21:2286–91. 7699630

[32] Wheaton L, Pope J. The minimally important difference for patient-reported outcomes in spondyloarthropathies including pain, fatigue, sleep, and health assessment questionnaire. J Rheumatol 2010;37:816–22. 20110516 10.3899/jrheum.090086

[33] Aissaoui N, Rostom S, Hakkou J et al Fatigue in patients with ankylosing spondylitis: prevalence and relationships with disease-specific variables, psychological status, and sleep disturbance. Rheumatol Int 2012;32:2117–24. 21516494 10.1007/s00296-011-1928-5

[34] Durmus D, Alayli G, Cil E, Canturk F. Effects of a home-based exercise program on quality of life, fatigue, and depression in patients with ankylosing spondylitis. Rheumatol Int 2009;29:673–7. 18985351 10.1007/s00296-008-0756-8

[35] Ibn Yacoub Y, Amine B, Laatiris A, Abouqal R, Hajjaj-Hassouni N. Assessment of fatigue in Moroccan patients with ankylosing spondylitis. Clin Rheumatol 2010;29:1295–9. 20803045 10.1007/s10067-010-1558-4

[36] Stebbings SM, Treharne GJ, Jenks K, Highton J. Fatigue in patients with spondyloarthritis associates with disease activity, quality of life and inflammatory bowel symptoms. Clin Rheumatol 2014;33:1467–74. 24322832 10.1007/s10067-013-2445-6

[37] Turan Y, Duruoz MT, Bal S et al Assessment of fatigue in patients with ankylosing spondylitis. Rheumatol Int 2007;27:847–52. 17252263 10.1007/s00296-007-0313-x

[38] Alkan BM, Fidan F, Erten S et al Fatigue and correlation with disease-specific variables, spinal mobility measures, and health-related quality of life in ankylosing spondylitis. Modern Rheumatol 2013;23:1101–7. 10.1007/s10165-012-0800-0 23224065

[39] Da Costa D, Zummer M, Fitzcharles MA. Biopsychosocial determinants of physical and mental fatigue in patients with spondyloarthropathy. Rheumatol Int 2011;31:473–80. 20091037 10.1007/s00296-009-1250-7

[40] Van Tubergen A, Coenen J, Landewé R et al Assessment of fatigue in patients with ankylosing spondylitis: a psychometric analysis. Arthritis Rheum 2002;47:8–16. 11932872 10.1002/art1.10179

[41] Gunaydin R, Karatepe AG, Cesmeli N, Kaya T. Fatigue in patients with ankylosing spondylitis: relationships with disease-specific variables, depression, and sleep disturbance. Clin Rheumatol 2009;28:1045–51. 19504231 10.1007/s10067-009-1204-1

[42] Revicki DA, Rentz AM, Luo MP, Wong RL. Psychometric characteristics of the short form 36 health survey and functional assessment of chronic illness Therapy-Fatigue subscale for patients with ankylosing spondylitis. Health Qual Life Outcomes 2011;9:36. 21600054 10.1186/1477-7525-9-36 PMC3124410

[43] Bedaiwi M, Sari I, Thavaneswaran A et al Fatigue in ankylosing spondylitis and nonradiographic axial spondyloarthritis: analysis from a longitudinal observation cohort. J Rheumatol 2015;42:2354–60. 26523020 10.3899/jrheum.150463

[44] Schneeberger EE, Marengo MF, Dal Pra F, Maldonado Cocco JA, Citera G. Fatigue assessment and its impact in the quality of life of patients with ankylosing spondylitis. Clin Rheumatol 2015;34:497–501. 24870035 10.1007/s10067-014-2682-3

[45] Wanders AJB, Gorman JD, Davis JC, Landewe RBM, Van Der Heijde DMFM. Responsiveness and discriminative capacity of the assessments in ankylosing spondylitis disease-controlling antirheumatic therapy core set and other outcome measures in a trial of etanercept in ankylosing spondylitis. Arthritis Care Res 2004;51:1–8. 10.1002/art.20075 14872448

[46] Dernis-Labous E, Messow M, Dougados M. Assessment of fatigue in the management of patients with ankylosing spondylitis. Rheumatology 2003;42:1523–8. 13130145 10.1093/rheumatology/keg421

[47] Streiner DL, Norman GR. Health measurement scales—a practical guide to their development and use. 4th edn Oxford, UK: Oxford University Press, 2008.

[48] Naegeli AN, Flood E, Tucker J, Devlen J, Edson-Heredia E. The patient experience with fatigue and content validity of a measure to assess fatigue severity: qualitative research in patients with ankylosing spondylitis (AS). Health Qual Life Outcomes 2013;11:192. 24215664 10.1186/1477-7525-11-192 PMC3832747

[49] Farren W, Goodacre L, Stigant M. Fatigue in ankylosing spondylitis: causes, consequences and self-management. Musculoskelet Care 2013;11:39–50. 10.1002/msc.1029 22825963

[50] Hewlett S, Cockshott Z, Byron M et al Patients' perceptions of fatigue in rheumatoid arthritis: overwhelming, uncontrollable, ignored. Arthritis Rheum 2005;53:697–702. 16208668 10.1002/art.21450

[51] Eilertsen G, Ormstad H, Kirkevold M et al Similarities and differences in the experience of fatigue among people living with fibromyalgia, multiple sclerosis, ankylosing spondylitis and stroke. J Clin Nursing 2015;24:2023–34. 10.1111/jocn.12774 25661994

[52] de Raaf PJ, de Klerk C, Timman R, Hinz A, van der Rijt CC. Differences in fatigue experiences among patients with advanced cancer, cancer survivors, and the general population. J Pain Sympt Manag 2012;44:823–30. 10.1016/j.jpainsymman.2011.12.279 22795903

[53] Whitehead L. The measurement of fatigue in chronic illness: a systematic review of unidimensional and multidimensional fatigue measures. J Pain Sympt Manag 2009;37:107–28. 10.1016/j.jpainsymman.2007.08.019 19111779

[54] Boers M, Kirwan JR, Wells G et al Developing core outcome measurement sets for clinical trials: OMERACT Filter 2.0. J Clin Epidemiol 2014;67:745–53. 24582946 10.1016/j.jclinepi.2013.11.013

[55] Staniszewska S, Haywood KL, Brett J, Tutton L. Patient and public involvement in patient-reported outcome measures: evolution not revolution. Patient 2012;5:79–87. 22428752 10.2165/11597150-000000000-00000

[56] US Food and Drug Administration. Guidance for industry: patient-reported outcome measures: use in medical product development to support labeling claims. Rockville: Department of Health and Human Services, Food and Drug Administration, Centre for Drug Evaluation and Research, 2009.

[57] Devlin N, Appleby J. Getting the most out of PROMs. Putting health outcomes at the heart of NHS decision-making. London: The Kings Fund. 2010 https://www.kingsfund.org.uk/sites/files/kf/Getting-the-most-out-of-PROMs-Nancy-Devlin-John-Appleby-Kings-Fund-March-2010.pdf (11 January 2018, date last accessed).

[58] Patrick DL, Burke LB, Powers JH et al Patient-reported outcomes to support medical product labeling claims: FDA perspective. Value Health 2007;10(Suppl 2):S125–37. 17995471 10.1111/j.1524-4733.2007.00275.x

