# Supplementary material for: Assessing fatigue in adults with axial spondyloarthritis: a systematic review of the quality and acceptability of patient-reported outcome measures
Source: Rheumatol Adv Pract. 2018 May 29;2(2):rky017. doi: 10.1093/rap/rky017 (PMC6649921; doi:10.1093/rap/rky017)
Supplement: Supplementary Data [file rky017_supp.docx]

**Appendix 1: Search Strategy**

Search 1 – PROM evaluation studies

Database: Ovid MEDLINE(R) <1946 to August Week 5 2017>, Embase Classic+Embase <1947 to 2017 September 08>, PsycINFO <1967 to September Week 1 2017>

Search Strategy:

--------------------------------------------------------------------------------

1 Spondylitis, Ankylosing/ or (Axial Spondyl* or Spondyl*, Axial or (Ankylosing Spondyl* or Spondyl*, Ankylosing) or Spondyloarthropath* or Spondyloarthritis).ti,ab. (49090)

2 (Fatigue or Asthenia* or Lassitude or Exhaust* or Inertia or Drows* or Letharg* or (Tiring or Tired* or Weary or Weariness)).mp. (544147)

3 (HR-PRO or HRPRO or HRQL or HRQoL or QL or QoL or (PRO or PROs or PROM or PROMs)).ti,ab. or (VAS or NRS).mp. or visual analogue scale*.mp. or numeric* rating scale*.mp. or quality of life.mp. or (health index* or health indices or health profile*).ti,ab. or health status.mp. or ((patient or self or proxy) adj (appraisal* or appraised or report or reported or reporting or rated or rating* or based or assessed or assessment*)).ti,ab. or ((disability or function or functional or functions or subjective or utility or utilities or wellbeing or well being) adj2 (index or indices or instrument or instruments or measure or measures or questionnaire* or profile or profiles or scale or scales or score or scores or status or survey or surveys)).ti,ab. (2092925)

4 (instrumentation or methods).sh. or (Validation Studies or Comparative Study).pt. or exp Psychometrics/ or psychometr*.ti,ab. or (clinimetr* or clinometr*).tw. or exp "Outcome Assessment (Health Care)"/ or outcome assessment.ti,ab. or outcome measure*.tw. or exp Observer Variation/ or observer variation.ti,ab. or exp Health Status Indicators/ or exp "Reproducibility of Results"/ or reproducib*.ti,ab. or exp Discriminant Analysis/ or (reliab* or unreliab* or valid* or coefficient or homogeneity or homogeneous or "internal consistency").ti,ab. or (cronbach* and (alpha or alphas)).ti,ab. or (item and (correlation* or selection* or reduction*)).ti,ab. or (agreement or precision or imprecision or "precise values" or test-retest).ti,ab. or (test and retest).ti,ab. or (reliab* and (test or retest)).ti,ab. or (stability or interrater or inter-rater or intrarater or intra-rater or intertester or inter-tester or intratester or intra-tester or interobserver or inter-observer or intraobserver or intraobserver or intertechnician or inter-technician or intratechnician or intra-technician or interexaminer or inter-examiner or intraexaminer or intra-examiner or interassay or interassay or intraassay or intra-assay or interindividual or inter-individual or intraindividual or intra-individual or interparticipant or inter-participant or intraparticipant or intra-participant or kappa or kappa's or kappas or repeatab*).ti,ab. or ((replicab* or repeated) and (measure or measures or findings or result or results or test or tests)).ti,ab. or (generaliza* or generalisa* or concordance).ti,ab. or (intraclass and correlation*).ti,ab. or (discriminative or "known group" or factor analysis or factor analyses or dimension* or subscale*).ti,ab. or (multitrait and scaling and (analysis or analyses)).ti,ab. or (item discriminant or interscale correlation* or error or errors or "individual variability").ti,ab. or (variability and (analysis or values)).ti,ab. or (uncertainty and (measurement or measuring)).ti,ab. or ("standard error of measurement" or sensitiv* or responsive*).ti,ab. or ((minimal or minimally or clinical or clinically) and (important or significant or detectable) and (change or difference)).ti,ab. or (small* and (real or detectable) and (change or difference)).ti,ab. or (meaningful change or "ceiling effect" or "floor effect" or "Item response model" or IRT or Rasch or "Differential item functioning" or DIF or "computer adaptive testing" or "item bank" or "cross-cultural equivalence").ti,ab. (12549272)

5 (addresses or biography or case reports or comment or directory or editorial or festschrift or interview or lectures or legal cases or legislation or letter or news or newspaper article or patient education handout or popular works or congresses or consensus development conference or consensus development conference, nih or practice guideline).pt. not (*animals/ not *humans/) (5263211)

6 (1 and 2 and 3 and 4) not 5 (347)

7 limit 6 to english language (341)

8 limit 7 to human (316)

9 limit 8 to yr="1980 -Current" (316)

***************************

Search 2 – Named measures search

Database: Ovid MEDLINE(R) <1946 to August Week 5 2017>, Embase Classic+Embase <1947 to 2017 September 08>, PsycINFO <1967 to September Week 1 2017>

Search Strategy:

--------------------------------------------------------------------------------

1 Spondylitis, Ankylosing/ or (Ankylosing Spondyl* or Spondyl*, Ankylosing or Spondyloarthropath* or Spondyloarthritis).ti,ab. (49014)

2 (Fatigue or Asthenia* or Lassitude or Exhaust* or Inertia or Drows* or Letharg* or (Tiring or Tired* or Weary or Weariness)).mp. (544147)

3 (Fatigue Assessment Scale or FAS or Fatigue Impact Scale or FIS or Fatigue Scale or FS or Fatigue Symptom Inventory or FSI or Myasthenia Gravis Fatigue Scale or Multidimensional Fatigue Symptom Inventory or Multi-dimensional Fatigue Symptom Inventory or MFSI or MFSI-SF or Parkinsons Fatigue Scale or (Pearson and Byars Fatigue Feeling Checklist) or Revised Piper Fatigue Scale or Piper Fatigue Scale or R-PFS or PFS or Rhoten Fatigue Scale or (Schedule of Fatigue and Anergia) or SOFA or Schwartz Cancer Fatigue Scale or SCFS or Visual Analog Fatigue Scale or VAS-F or Checklist Individual Strength or CIS20R or CIS8R or Multidimensional Fatigue Scale or Multi-dimensional Fatigue Scale or MFS or Fatigue Questionnaire or Multidimensional Assessment of Fatigue or Multi-Dimensional Assessment of Fatigue or MAF or Multi-dimensional Health Assessment Questionnaire or Multidimensional Health Assessment Questionnaire or MDHAQ or Profile of Fatigue or ProF or Multidimensional Fatigue Inventory or Multi-dimensional Fatigue Inventory or MFI or Bath Ankylosing Spondylitis Disease Activity Index or BASDAI or (mini adj BASDAI) or ((Bristol Rheumatoid Arthritis Fatigue Multidimensional Questionnaire adj MDQ) or NRS) or ((Bristol Rheumatoid Arthritis Fatigue Multi-dimensional Fatigue Questionnaire adj MDQ) or NRS) or ((BRAF adj MDQ) or NRS) or BRAFMDQ or BRAFNRS or Short-Form Health Survey or SF-36 or SF36 or SF36-V2 or SF36V2 or Bath Ankylosing Spondylitis Functional Index or BASFI or Evaluation of Ankylosing Spondylitis Quality of Life or EASi-QoL or EASiQoL or Brief Fatigue Inventory or BFI or Functional Assessment of Chronic Illness Therapy or (FACIT adj (F or Fatigue)) or Evaluation of Daily Activity Questionnaire or EDAQ or Worst Fatigue-Numeric Rating Scale or WF-NRS or WFNRS or Fatigue Severity Scale or FSS or Chalder Fatigue Scale or CFS or Patient Reported Outcome* Measurement Information System or PROMIS Visual Analogue Scale* or VAS or Numeric Rating Scale* or NRS).mp. (436787)

4 (instrumentation or methods).sh. or (Validation Studies or Comparative Study).pt. or exp Psychometrics/ or psychometr*.ti,ab. or (clinimetr* or clinometr*).tw. or exp "Outcome Assessment (Health Care)"/ or outcome assessment.ti,ab. or outcome measure*.tw. or exp Observer Variation/ or observer variation.ti,ab. or exp Health Status Indicators/ or exp "Reproducibility of Results"/ or reproducib*.ti,ab. or exp Discriminant Analysis/ or (reliab* or unreliab* or valid* or coefficient or homogeneity or homogeneous or "internal consistency").ti,ab. or (cronbach* and (alpha or alphas)).ti,ab. or (item and (correlation* or selection* or reduction*)).ti,ab. or (agreement or precision or imprecision or "precise values" or test-retest).ti,ab. or (test and retest).ti,ab. or (reliab* and (test or retest)).ti,ab. or (stability or interrater or inter-rater or intrarater or intra-rater or intertester or inter-tester or intratester or intra-tester or interobserver or inter-observer or intraobserver or intraobserver or intertechnician or inter-technician or intratechnician or intra-technician or interexaminer or inter-examiner or intraexaminer or intra-examiner or interassay or interassay or intraassay or intra-assay or interindividual or inter-individual or intraindividual or intra-individual or interparticipant or inter-participant or intraparticipant or intra-participant or kappa or kappa's or kappas or repeatab*).ti,ab. or ((replicab* or repeated) and (measure or measures or findings or result or results or test or tests)).ti,ab. or (generaliza* or generalisa* or concordance).ti,ab. or (intraclass and correlation*).ti,ab. or (discriminative or "known group" or factor analysis or factor analyses or dimension* or subscale*).ti,ab. or (multitrait and scaling and (analysis or analyses)).ti,ab. or (item discriminant or interscale correlation* or error or errors or "individual variability").ti,ab. or (variability and (analysis or values)).ti,ab. or (uncertainty and (measurement or measuring)).ti,ab. or ("standard error of measurement" or sensitiv* or responsive*).ti,ab. or ((minimal or minimally or clinical or clinically) and (important or significant or detectable) and (change or difference)).ti,ab. or (small* and (real or detectable) and (change or difference)).ti,ab. or (meaningful change or "ceiling effect" or "floor effect" or "Item response model" or IRT or Rasch or "Differential item functioning" or DIF or "computer adaptive testing" or "item bank" or "cross-cultural equivalence").ti,ab. (12549272)

5 1 and 2 and 3 and 4 (319)

6 limit 5 to english language (312)

7 limit 6 to human (288)

8 limit 7 to yr="1980 -Current" (288)

9 remove duplicates from 8 (220)

***************************

**Appendix 2: Quality criteria for the assessment of reported measurement properties (1, 2)**

| **Measurement properties** | **Rating** | **Quality criteria** |
| --- | --- | --- |
| **Validity** |  |  |
| Content validity | + | Authors provide a clear description of the measurement aim, target population, concept(s) measured and process of item selection.  Members of the target population and experts in the field were clearly identified as being involved in development. For measures applied  for the first time in a new population, evidence that the views of members of the target population (and experts in the field) have been  sought to determine relevance, comprehension and comprehensiveness. |
|  | ? | Insufficient evidence available |
|  | - | No detail re measurement aim, target population, concept(s) measured, process of item selection; members of the target population or  experts were not specifically involved in development.  For measures applied for the first time in a new population, evidence whereby the relevance and acceptability of the measure with  members of the target audience or experts was not provided. |
|  |  |  |
| Construct validity – Structural validity | + | Factors should explain at least 50% of the variance |
|  | ? | Explained variance not stated |
|  | - | Factors explain <50% of the variance |
|  |  |  |
| Construct validity – Hypothesis testing | + | Correlations with measures of the same construct should be >0.50 OR at least 75% of the results in accordance with hypothesized  associations AND correlations with related constructs should be higher than with those reported with unrelated constructs |
|  | ? | Only report correlations with unrelated constructs OR the extent to which between group differences are expected is not described /  justified |
|  | - | Correlations with measures of the same construct are <0.50 OR < 75% of the results in accordance with hypothesized associations OR  correlations with related constructs are lower than those reported with unrelated constructs |
|  |  |  |
| Construct validity – Known-group validity  (not part of the COSMIN checklist) | + | Hypothesised between group differences are supported (or can be assumed) AND between group differences are statistically significant |
|  | ? | Between group differences are poorly hypothesized, but between group differences are statistically significant |
|  | - | Expected between group difference poorly defined or justified AND the statistical significance of between group differences not reported |
|  |  |  |
| **Reliability** |  |  |
| Internal consistency | + | Cronbach’s alpha(s) ≥ 0.70 |
|  | ? | Cronbach’s alpha not determined or dimensionality unknown |
|  | - | Cronbach’s alpha(as) < 0.70 |
|  |  |  |
| Reliability (test-retest / inter-rater / intra-rater) | + | Intra-class Correlation Coefficient (ICC)/ weighted Kappa ≥0.70 OR Pearson’s r ≥0.80 |
|  | ? | Neither ICC/ weighted Kappa, nor Pearson’s r established |
|  | - | ICC/ weighted Kappa <0.70 OR Pearson’s r <0.80 |
|  |  |  |
| Reliability – measurement error | N/A | Descriptive (not rated) |
|  |  |  |
| Responsiveness | + | Change-score correlations with measures of the same construct are >0.50 OR at least 75% of the results are in accordance with  hypothesized associations OR the Area Under the Curve (AUC) is >0.70 AND change-score correlations with measures of related constructs  are higher than those reported with unrelated constructs |
|  | ? | Solely correlations with unrelated constructs |
|  | - | Change-score correlations with measure of the same construct <0.50 OR < 75% of the results are in accordance with hypothesized  associations OR AUC is <0.70 AND change-score correlations with related constructs are lower than those reported with unrelated  constructs |
|  |  |  |
| Interpretability | N/A | Descriptive (not rated) - requires evidence that the minimal important (within-person) change (MIC) and/or minimal importance (between  group) difference (MID) exceeds evidence of the smallest detectable difference (SDD). Supported by evidence of acceptable data quality  (score distribution, absence of end effects (floor/ ceiling) |

**Appendix 3: Characteristics of included studies (*n=23*)**

| **Study** | **Country** | **PROM(s) evaluated** | **Sample** | **Setting** | **Study focus** |
| --- | --- | --- | --- | --- | --- |
| Aissaoui et al. (3) | Morocco | MAF  10cm VAS | AS patients  Mean age 38.52, 68% male | **Not reported** | Cross-sectional study evaluating the frequency of fatigue, and its relationship with other variables (disease-specific variables, psychological status and sleep disturbance) in Moroccan patients diagnosed with AS. |
| Bedaiwi et al. (4) | Canada | FSS | AS patients and Nr-AxSpa  Mean age 40.5, 71.9% male | Rheumatology clinic | Longitudinal cohort study investigating fatigue prevalence, associated factors, and the impact of tumour necrosis factor inhibitors (TNFi) on a patient subgroup. Study was conducted in Canada with patients diagnosed with AS or Nr-AxSpa. |
| Bodur et al. (5) | Turkey | SF-36 vitality | AS patients  Mean age 39.4, 76.1% male | **Not reported** | Cross-sectional study evaluating quality of life in Turkish patients diagnosed with AS. |
| Da Costa et al. (6) | Canada | MFI-20 | SpA patients  Mean age 46.5, 46.4% male | 3 university affiliated rheumatology sites and 1 satellite community clinic | Cross-sectional study investigating contributors to dimensions of fatigue. Study was conducted in Canada with SpA patients. |
| Dagfinrud et al. (7) | Norway | 10cm VAS  SF-36 vitality | AS patients  Mean age 47, 58% male | Rheumatology clinic^a^ | Cross-sectional study investigating levels of fatigue, the relationship between fatigue and other factors (demographics, self-reported, clinician measures) and the performance of a generic and a disease-specific measure of fatigue. Study conducted in Norway with AS patients. |
| Dernis-Labous et al. (8) | France | 10cm VAS | AS patients  Mean age 41.7, 68% male | Rheumatology clinic | Review of evidence from two double-blind, placebo-controlled RCT’s evaluating fatigue prevalence and the clinical relevance and effect of NSAID therapy. Study was conducted with French AS patients. |
| Durmus et al. (9) | Turkey | MAF  SF-36 vitality | AS patients  Mean age **Not reported**, % male **Not reported** | **Not reported** | Trial study investigating the effect of a home-based exercise programme on QoL. Study used Turkish AS patients. |
| Fallahi et al. (10) | Iran | 10cm VAS | AS patients  Mean age 37.74, 73.62% male | Rheumatology clinic | Cross-sectional study evaluating the reliability of the Persian version of the ASQoL questionnaire. Study conducted with Iranian AS patients. |
| Fernandez-Sueiro et al. (11) | Spain | 10cm VAS | PsA, Axial PsA and AS patients  Mean age **Not reported**, % male **Not reported** | AS clinic | Longitudinal study conducted in Spain evaluating the validity of BASDAI for use in PsA. AxSpa and AS patients were recruited to form comparator/ control groups and reported separately. |
| Gunaydin et al. (12) | Turkey | 10cm VAS  MFSI-SF | AS patients  Mean age 39.6, 83.87% male | Outpatient clinic | Cross-sectional study evaluating fatigue frequency and its multi-dimensional structure, and its association with other variables (demographics, disease-specific). Study conducted with Turkish AS patients. |
| Ibn Yacoub et al. (13) | Morocco | 10cm VAS  MAF | AS patients  Mean age 38, 67% male | Department of Rheumatology of El Ayachi Hospital | Cross-sectional study investigating the aspects of fatigue and relationships with disease-specific variables of activity and severity. Study conducted with Moroccan AS patients. |
| Maksymowych et al. (14) | Canada | FACIT-Fatigue | AS patients  Mean age 45.7, 74.9% male | Rheumatology clinic^a^ | Cross-sectional postal survey exploring contributors to PASS and validating the PASS concept. Study conducted with Canadian AS patients. |
| Naegeli et al. (15) | US | BFI (worst fatigue item) | AS patients  Mean age 47, 38.4% male | **Not reported** | Cross-sectional qualitative study exploring the development and content validity of the WF-NRS. Study conducted with US AS patients. |
| Park et al. (16) | Korea | 10cm VAS | AS patients  Mean age 30, 92% male | Rheumatology clinic | Cross-sectional study evaluating the validity and reliability of a Korean translation of the BASDAI. Study conducted with Korean AS patients. |
| Revicki et al. (17) | US, Europe and Canada | FACIT-Fatigue  SF-36 vitality  10cm VAS | AS patients  Mean age 42, 75.8% male | Rheumatology clinic^b^ | A review of 2 RCT studies to evaluate the measurement properties of the SF-36 and FACIT-F in AS patients. Patients recruited from US, Europe and Canada. |
| Schneeberger et al. (18) | Argentina | 10cm VAS  FSS | AS patients  Mean age **Not reported**, % male **Not reported** | **Not reported** | Case-control study assessing fatigue prevalence and establishing main associated factors. Study conducted with Argentinian AS patients. |
| Stebbings et al. (19) | New Zealand | 10cm VAS  MAF | AxSpa patients  Mean age 43.42, 67% male | Rheumatology clinic in hospital | Cross-sectional study assessing fatigue severity, the performance of two different fatigue measures amd examine disease variables that may influence fatigue severity. Study conducted with AS patients in New Zealand. |
| Turan et al. (20) | Turkey | MAF  SF-36 | AS patients  Mean age 37.7, 82.35% male | Outpatients clinic | Cross-sectional study investigating the prevalence of fatigue and the relationship with other variables (clinical, functional parameters). Study conducted with Turkish AS patients. |
| van Tubergen et al. (21) | The Netherlands | MFI  10cm VAS  SF-36 vitality | AS patients  Mean age **Not reported**, % male **Not reported** | Rheumatology and outpatient’s clinic | PROM evaluation study using patients recruited from three sources. Also seeking to identify factors that influence fatigue and the association of fatigue with quality of life. Patients were Dutch with a diagnosis of AS. |
| Wanders et al. (22) | The Netherlands | FSS  SF-36 vitality | AS patients  Mean age **Not reported**, % male **Not reported** | University hospital | Double-blind clinical trial investigating the responsiveness and discriminative ability of, and relationships between, ASAS-recommended measures for the DC-ART core set. The study used Dutch AS patients. |
| Wheaton et al. (23) | Canada | Modified 10cm VAS | SpA patients  Mean age 44.7, 69.3% male | Hospital Rheumatology clinic | PROM evaluation study investigating the MID in SpA. The study used Canadian SpA patients. |
| Yilmaz et al. (24) | Turkey | 10cm VAS  SF-36 vitality | AS patients  Mean age 39.91, 79.72% male | Outpatients clinic | Cross-sectional study evaluating HR-QoL and to assess peripheral involvement impact on HR-QoL domains. The study used Turkish AS patients. |
| Alkan et al. (25) | Turkey | MAF | AS patients  Mean age **Not reported**, % male **Not reported** | Outpatient clinic | Cross-sectional study evaluating fatigue and its relationship with other variables (disease-specific, spinal mobility, HR-QoL). The study used Turkish AS patients. |

**Appendix 4: Methodological quality (COSMIN ^a^) per study (*n*=23) per PROM (*n*=9) and investigated measurement properties ^b^.**

| **PROM / Study** | **Country (language)** | **(n)** | **Reliability** | | | **Validity** | | | | **Responsiveness** | | **Interpretability** |
| --- | --- | --- | --- | --- | --- | --- | --- | --- | --- | --- | --- | --- |
|  |  |  | **Internal consistency** | **Reliability** | **Measurement error** | **Content validity** | **Structural validity** | **Hypothesis testing** | ***Known-groups*** | **Responsiveness (COSMIN)** | **Responsiveness - other** |  |
| **Multidimensional fatigue measures (3/9)** | | | | | | | | | | | | |
| ***MAF*** | |  |  |  |  |  |  |  |  |  |  |  |
| Aissaoui et al. (3) | Morocco  Arabic | 110 |  |  |  |  |  |  | *Poor* |  |  |  |
| Durmus et al. (9) | Turkey  Turkish | 43 |  |  |  |  |  |  | *Poor* |  |  |  |
| Ibn Yacoub et al. (13) | Morocco  Arabic | 100 |  |  |  |  |  | Poor |  |  |  |  |
| Stebbings et al. (19) | New Zealand  English | 67 |  |  |  |  |  | Poor |  |  |  |  |
| Turan et al. (20) | Turkey  Turkish | 68 |  |  |  |  |  | Poor |  |  |  |  |
| Alkan et al. (25) | Turkey  Turkish | 110 |  |  |  |  |  | Poor |  |  |  |  |
| ***MFI-20*** | |  |  |  |  |  |  |  |  |  |  |  |
| Da Costa et al. (6) | Canada  English (US) | 125 | Fair |  |  |  |  |  |  |  |  |  |
| van Tubergen et al. (21) | The Netherlands  Dutch | 40  *Sub-group – Arm 1* |  | Fair |  |  |  |  |  |  | *Mean (SD), ES, SRM, Guyatt* |  |
|  | The Netherlands  Dutch | 812  *Whole population* |  |  |  |  |  | Poor |  |  |  |  |
|  | The Netherlands  Dutch | 776  *Patients with BASDAI fatigue VAS score of 5 excluded* |  |  |  |  |  |  | *Poor* |  |  |  |
| ***MFSI-SF*** | |  |  |  |  |  |  |  |  |  |  |  |
| Gunaydin et al. (12) | Turkey  Turkish | 63 |  |  |  |  |  | Poor | *Poor* |  |  |  |
| **Unidimensional fatigue measures (2/9)** | | | | | | | | | | | | |
| ***FACIT-fatigue*** | |  |  |  |  |  |  |  |  |  |  |  |
| Revicki et al. (17) | Canada  English | 82 | Poor |  |  |  |  |  |  |  |  |  |
|  | US, Europe and Canada  English | 397 |  |  |  |  |  | Good |  |  |  |  |
| Maksymowych et al. (14) | Canada  English (US) | 302 |  |  |  |  |  |  |  | Poor |  |  |
| ***FSS^f^*** | |  |  |  |  |  |  |  |  |  |  |  |
| Bedaiwi et al. (4) | Canada  English (US) | 457 |  |  |  |  |  | Poor |  |  |  |  |
| Schneeberger et al. (18) | Argentina  Spanish | 159 |  |  |  |  |  | Poor | *Poor* |  |  |  |
| Wanders et al. (22) | The Netherlands  Dutch | 40 |  |  |  |  |  |  |  |  | *Mean (SD), mean change, ES, SRM, T-statistic* |  |
| **Single-item fatigue measures (3/9) – WF-NRS separately appraised (qualitative study) (WF-NRS a single item taken from BFI, but practical properties reviewed ONLY)** | | | | | | | | | | | | |
| ***10cm VAS (BASDAI)*** | |  |  |  |  |  |  |  |  |  |  |  |
| van Tubergen et al. (21) | The Netherlands  Dutch | 40 |  | Fair |  |  |  |  |  |  |  |  |
|  | The Netherlands  Dutch | 812 |  |  |  |  |  | Poor |  |  |  |  |
| Aissaoui et al. (3) | Morocco  Arabic | 110 |  |  |  |  |  | Poor |  |  |  |  |
| Dernis-Labous et al. (8) | France  French | 639 |  |  |  |  |  | Poor |  |  | *SRM only* |  |
| Fallahi et al. (10) | Iran  Persian | 163 |  |  |  |  |  | Poor |  |  |  |  |
| Gunaydin et al. (12) | Turkey  Turkish | 63 |  |  |  |  |  | Poor |  |  |  |  |
| Ibn Yacoub et al. (13) | Morocco  Moroccan | 100 |  |  |  |  |  | Poor |  |  |  |  |
| Park et al. (16) | Korea  Korean | 50 |  |  |  |  |  | Poor |  |  |  | Poor |
| Revicki et al. (17) | US, Europe and Canada  English | 397 |  |  |  |  |  | Good |  |  |  |  |
| Schneeberger et al. (18) | Argentina  Spanish | 159 |  |  |  |  |  | Poor |  |  |  |  |
| Stebbings et al. (19) | New Zealand  English | 67 |  |  |  |  |  | Poor |  |  |  |  |
| Yilmaz et al. (24) | Turkey  Turkish | 74 |  |  |  |  |  | Poor | *Poor* |  |  |  |
| Alkan et al. (25) | Turkey  Turkish | 110 |  |  |  |  |  | Poor |  |  |  |  |
| Dagfinrud et al. (7) | Norway  Norwegian | 302 |  |  |  |  |  |  |  | Fair |  |  |
| van Tubergen et al. (21) | Austria | 40 |  |  |  |  |  |  |  |  | *Mean (SD), ES, SRM, Guyatt* |  |
| Fernandez-Sueiro et al. (11) | Spain  Spanish | 103 |  |  |  |  |  |  |  |  |  | Poor |
| ***Modified 10cm VAS*** | |  |  |  |  |  |  |  |  |  |  |  |
| Wheaton et al. (23) | Canada  English (US) | 140 |  |  |  |  |  |  |  |  |  | Poor |
| **Fatigue-specific PROM subscale (1/9)** | | | | | | | | | | | | |
| ***SF-36 vitality subscale*** | |  |  |  |  |  |  |  |  |  |  |  |
| Revicki et al. (17) | US and Europe  English | 397 | Good |  |  |  |  | Fair |  |  |  |  |
| Bodur et al. (5) | Turkey  Turkish | 962 |  |  |  |  |  | Poor |  |  |  |  |
| Durmus et al. (9) | Turkey  Turkish | 43 |  |  |  |  |  |  | *Poor* |  |  |  |
| Turan et al. (20) | Turkey  Turkish | 68 |  |  |  |  |  | Poor |  |  |  |  |
| van Tubergen et al. (21) | The Netherlands  Dutch | 812 |  |  |  |  |  | Poor |  |  |  |  |
|  | The Netherlands  Dutch | 776 |  |  |  |  |  |  | *Poor* |  |  |  |
| Yilmaz et al. (24) | Turkey  Turkish | 74 |  |  |  |  |  | Poor | *Poor* |  |  |  |
| Alkan et al. (25) | Turkey  Turkish | 110 |  |  |  |  |  | Poor |  |  |  |  |
| Wanders et al. (22) | Netherlands  Dutch | 40 |  |  |  |  |  |  |  |  | *Mean (SD), mean change, ES, SRM* |  |

**Footnote:**

^a^ COSMIN provides a grading system for four possible rating outcomes: Excellent / Good / Fair / Poor (21, 22).

^b^ Measurement property quality has four possible rating outcomes (19, 23):

- adequate (+) - it fulfils the assessment criteria
- inadequate (-) - it has failed to meet the assessment criteria
- conflicting (+/-) – the evidence is conflicting and therefore difficult to interpret
- unclear (?) – the results are unclear

**References**

1. Terwee CB, Bot SD, de Boer MR, van der Windt DA, Knol DL, Dekker J, et al. Quality criteria were proposed for measurement properties of health status questionnaires. Journal of clinical epidemiology. 2007;60(1):34-42.

2. Conijn AP, Jens S, Terwee CB, Breek JC, Koelemay MJ. Assessing the quality of available patient reported outcome measures for intermittent claudication: a systematic review using the COSMIN checklist. European journal of vascular and endovascular surgery : the official journal of the European Society for Vascular Surgery. 2015;49(3):316-34.

3. Aissaoui N, Rostom S, Hakkou J, Berrada Ghziouel K, Bahiri R, Abouqal R, et al. Fatigue in patients with ankylosing spondylitis: prevalence and relationships with disease-specific variables, psychological status, and sleep disturbance. Rheumatology International. 2012;32(7):2117-24.

4. Bedaiwi M, Sari I, Thavaneswaran A, Ayearst R, Haroon N, Inman RD. Fatigue in Ankylosing Spondylitis and Nonradiographic Axial Spondyloarthritis: Analysis from a Longitudinal Observation Cohort. Journal of Rheumatology. 2015;42(12):2354-60.

5. Bodur H, Ataman Ş, Rezvani A, Buğdaycı DS, Çevik R, Birtane M, et al. Quality of life and related variables in patients with ankylosing spondylitis. Quality of Life Research: An International Journal of Quality of Life Aspects of Treatment, Care & Rehabilitation. 2011;20(4):543-9.

6. Da Costa D, Zummer M, Fitzcharles MA. Biopsychosocial determinants of physical and mental fatigue in patients with spondyloarthropathy. Rheumatology International. 2011;31(4):473-80.

7. Dagfinrud H, Vollestad NK, Loge JH, Kvien TK, Mengshoel AM. Fatigue in patients with ankylosing spondylitis: A comparison with the general population and associations with clinical and self-reported measures. Arthritis & Rheumatism-Arthritis Care & Research. 2005;53(1):5-11.

8. Dernis-Labous E, Messow M, Dougados M. Assessment of fatigue in the management of patients with ankylosing spondylitis. Rheumatology. 2003;42(12):1523-8.

9. Durmus D, Alayli G, Cil E, Canturk F. Effects of a home-based exercise program on quality of life, fatigue, and depression in patients with ankylosing spondylitis. Rheumatology International. 2009;29(6):673-7.

10. Fallahi S, Jamshidi AR, Bidad K, Qorbani M, Mahmoudi M. Evaluating the reliability of Persian version of ankylosing spondylitis quality of life (ASQoL) questionnaire and related clinical and demographic parameters in patients with ankylosing spondylitis. Rheumatology International. 2014;34(6):803-9.

11. Fernandez-Sueiro JL, Willisch A, Pertega-Diaz S, Tasende JAP, Fernandez-Lopez JC, Villar NO, et al. Validity of the Bath Ankylosing Spondylitis Disease Activity Index for the evaluation of disease activity in axial psoriatic arthritis. Arthritis Care and Research. 2010;62(1):78-85.

12. Gunaydin R, Karatepe AG, Cesmeli N, Kaya T. Fatigue in patients with ankylosing spondylitis: relationships with disease-specific variables, depression, and sleep disturbance. Clinical Rheumatology. 2009;28(9):1045-51.

13. Ibn Yacoub Y, Amine B, Laatiris A, Abouqal R, Hajjaj-Hassouni N. Assessment of fatigue in Moroccan patients with ankylosing spondylitis. Clinical Rheumatology. 2010;29(11):1295-9.

14. Maksymowych WP, Richardson R, Mallon C, van der Heijde D, Boonen A. Evaluation and validation of the patient acceptable symptom state (PASS) in patients with ankylosing-spondylitis. Arthritis & Rheumatism-Arthritis Care & Research. 2007;57(1):133-9.

15. Naegeli AN, Flood E, Tucker J, Devlen J, Edson-Heredia E. The patient experience with fatigue and content validity of a measure to assess fatigue severity: Qualitative research in patients with ankylosing spondylitis (AS). Health and Quality of Life Outcomes. 2013;11 (1) (no pagination)(192).

16. Park HJ, Kim S, Lee JE, Jun JB, Bae SC. The reliability and validity of a Korean translation of the BASDAI in Korean patients with Ankylosing spondylitis. Value in Health. 2008;11:S99-S104.

17. Revicki DA, Rentz AM, Luo MP, Wong RL. Psychometric characteristics of the short form 36 health survey and functional assessment of chronic illness Therapy-Fatigue subscale for patients with ankylosing spondylitis. Health Qual Life Outcomes. 2011;9:36.

18. Schneeberger EE, Marengo MF, Dal Pra F, Maldonado Cocco JA, Citera G. Fatigue assessment and its impact in the quality of life of patients with ankylosing spondylitis. Clinical Rheumatology. 2015;34(3):497-501.

19. Stebbings SM, Treharne GJ, Jenks K, Highton J. Fatigue in patients with spondyloarthritis associates with disease activity, quality of life and inflammatory bowel symptoms. Clinical Rheumatology. 2014;33(10):1467-74.

20. Turan Y, Duruoz MT, Bal S, Guvenc A, Cerrahoglu L, Gurgan A. Assessment of fatigue in patients with ankylosing spondylitis. Rheumatology International. 2007;27(9):847-52.

21. Van Tubergen A, Coenen J, Landewé R, Spoorenberg A, Chorus A, Boonen A, et al. Assessment of Fatigue in Patients With Ankylosing Spondylitis: A Psychometric Analysis. Arthritis & Rheumatism: Arthritis Care & Research. 2002;47(1):8-16.

22. Wanders AJB, Gorman JD, Davis JC, Landewe RBM, Van Der Heijde DMFM. Responsiveness and Discriminative Capacity of the Assessments in Ankylosing Spondylitis Disease-Controlling Antirheumatic Therapy Core Set and Other Outcome Measures in a Trial of Etanercept in Ankylosing Spondylitis. Arthritis Care and Research. 2004;51(1):1-8.

23. Wheaton L, Pope J. The Minimally Important Difference for Patient-reported Outcomes in Spondyloarthropathies including Pain, Fatigue, Sleep, and Health Assessment Questionnaire. Journal of Rheumatology. 2010;37(4):816-22.

24. Yilmaz O, Tutoglu A, Garip Y, Ozcan E, Bodur H. Health-related quality of life in Turkish patients with ankylosing spondylitis: impact of peripheral involvement on quality of life in terms of disease activity, functional status, severity of pain, and social and emotional functioning. Rheumatology International. 2013;33(5):1159-63.

25. Alkan BM, Fidan F, Erten S, Aksekili H, Alemdar A, Eroglu E, et al. Fatigue and correlation with disease-specific variables, spinal mobility measures, and health-related quality of life in ankylosing spondylitis. Modern Rheumatology. 2013;23(6):1101-7.
